# Stress and strain relations of RC circular, square and rectangular columns externally wrapped with fiber ropes

**DOI:** 10.1038/s41598-024-54586-9

**Published:** 2024-02-20

**Authors:** Qudeer Hussain, Anat Ruangrassamee, Tidarut Jirawattanasomkul, Dawei Zhang

**Affiliations:** 1 Center of Excellence in Earthquake Engineering and Vibration, Department of Civil Engineering, Faculty of Engineering, Chulalongkorn University, Bangkok, Thailand; 2 Center of Excellence in Innovative Construction Materials, Department of Civil Engineering, Faculty of Engineering, Chulalongkorn University, Bangkok, Thailand; 3 College of Civil Engineering and Architecture, Zhejiang University, Hangzhou, China

**Keywords:** Hemp confinement, Cotton confinement, FRRP, Analytical models, Compressive behavior, Reinforced concrete, Engineering, Civil engineering

## Abstract

This study explores the potential use of low-cost natural fiber reinforced rope polymers (FRRP) to improve the compressive behavior of circular, square, and rectangular reinforced concrete (RC) specimens. A total of 42 specimens were tested under monotonic axial compression in three groups. Groups were formed to differentiate specimens with different cross-sectional shapes such as circular, square, and rectangular. The findings demonstrate that FRRP can effectively boost the compressive behavior of RC columns. Circular specimens with three-layer hemp FRRP exhibited a 200% increase in compressive strength and a 270% improvement in corresponding strain. Cotton FRRP provided a 117% boost in compressive strength and a 233% enhancement in strain. In square specimens, three-layer hemp FRRP resulted in a 110% rise in compressive strength and a 186% increase in strain, while cotton confinement yielded improvements of 95% and 144%, respectively. For the square and rectangular specimens, the improvement in the compressive behavior was reduced compared to the circular specimens because of stress concentrations near corners. Moreover, the study showed that the hemp FRRP confinement outperformed the cotton confinement. The investigation also revealed that the existing analytical models were inadequate in predicting the mechanical properties of RC confined with natural FRRP. Therefore, the study introduces novel equations to predict the compressive strength and corresponding strain for both hemp and cotton confined concrete in various cross-sectional types. These proposed equations exhibit a good level of accuracy in predicting the compressive strength and corresponding strain.

## Introduction

Massive impairments of reinforced concrete (RC) structures have been reported in recent earthquakes attributed to their seismic vulnerability^1,2^. This is because the older building codes (e.g., pre-1973) did not incorporate proper seismic detailing procedures like those found in the current codes^3,4^. Apart from the catastrophic failures faced in the past, many existing structures contain the same substandard detailing and need quick repairs to their seismically prone regions^5–8^. An earthquake of intensity Mw 6.1 occurred in northern Thailand on 5th May 2014 that caused severe damage to many existing buildings ascribed to the poor detailing and structural design of those buildings^9^. Thus, repairing and rehabilitation of existing substandard structures becomes vital. The conventional techniques to strengthen the deficient RC members include the external wrapping of the potential plastic hinge regions using concrete^10^ or steel jackets^11^. The key disadvantages of these techniques include labor-intensive and time-consuming^12–14^. Further, the concrete jacketing results in enlarged sections and imparts noticeable weight to the structure, whereas the steel jacketing is prone to inner surface corrosion and involves difficulty in its handling^15^. Due to the resulting enlarged sections and noticeable added weight, the stiffness of the structures may be altered^13,16^. Given this, fiber-reinforced polymer (FRP) jackets have emerged as an efficient alternative to these conventional jacketing techniques. The advantages of using fiber reinforced polymers (FRP) in construction are their low weight and high strength-to-weight ratio, easy and quick installation, resistance to corrosion, and durability^17^. FRP jackets have been found to offer numerous advantages over traditional strengthening methods, including high durability, corrosion resistance, ease and speed of application, and a favorable strength-to-weight ratio. Additionally, these jackets have demonstrated their capability to enhance the strength of substandard reinforced concrete elements^18–23^. Two key issues are linked with these synthetic FRP jackets, (1) FRP jackets are costly^24,25^ and might not support their use for small-scale strengthening works, and (2) the fabrication process of the synthetic FRP jackets involves the use of chemicals that are detrimental to the skin and may cause irritant and allergic contact dermatitis^26^.

The use of natural FRP as an alternative to synthetic FRP jackets has been investigated in recent years^27–30^. The substantial reduction in the cost of the natural FRPs in comparison to that of the synthetic FRPs is among their salient advantages^31,32^. Yooprasertchai et al.^27^ performed a study to evaluate the effectiveness of natural fibers such as sisal and jute in enhancing the performance of shear-deficient RC columns. The results of the study showed that both types of fibers improved the performance of shear-dominated RC columns to a level comparable to that of RC columns strengthened with synthetic FRP jackets. Suparp et al.^33^ conducted a study to investigate the efficacy of hemp confinement in confining lightweight aggregate concrete and improving its compressive strength and ductility. The study found that by increasing layers of hemp fiber ropes, an expressive enhancement in the compressive strength and corresponding strain of the lightweight aggregate concrete was achieved. Hussain et al.^34^ utilized hemp, cotton, and polyester fiber ropes to enhance the ultimate strength, strain, and deformability of the concrete. The compressive stress versus strain response of the concrete was significantly improved after the confinement, irrespective of the confining fiber type. Hussain et al.^35^ confined square concrete columns with hemp, cotton, and polyester fiber ropes to improve their compressive strength and ductility. They concluded that the corner radius size employed for improving the efficiency of external wraps directly influenced the peak compressive strengths and the corresponding strains.

So far, the use of natural fibers in increasing the compressive strength and ductility has been limited to plain concrete. Previous research has indicated that plain concrete confined with FRP behaves differently than concrete confined with steel.^36–38^. Consequently, the design recommendations for one type of confinement cannot be extended to the other^39^. Previous research has investigated and put forward analytical models concerning the ultimate compressive strength and strain of plain concrete confined with natural fiber rope reinforced polymer (FRRP)^33–35,40^. However, it is worth noting that none of these studies have specifically addressed the predictive capability of these analytical models for the ultimate compressive strength and strain of reinforced concrete.

Recently, hemp confinement was utilized to enhance the compressive behavior of concrete made with brick waste^41^. Jirawattanasomkul et al.^42^ utilized jute, hemp, and cotton confinement to enhance mechanical properties of natural aggregate concrete. Hussain et al.^34^ enhanced the compressive behavior of plain concrete by using cotton ropes and presented promising results. However, none of these studies considered the presence of steel reinforcement in the concrete. Therefore, the present study aims to extend the application of the natural hemp and cotton fiber ropes to confine the reinforced concrete and assess their efficacy in the presence of internal steel confinement. Further, this study investigates the performance of the existing stress–strain models for FRP confined reinforced concrete in predicting the peak strength and strain of the natural FRP confined reinforced concrete. By noticing the inability of existing models to predict the compressive behavior of hemp and cotton confined reinforced concrete, the analytical work was extended to propose expressions based on nonlinear regression. In addition, a design-oriented approach was developed to trace the complete compressive stress versus strain behavior of reinforced concrete confined with hemp and cotton confinement.

## Experimental program

### Test matrix

Experimental program was conducted on 42 RC specimens divided mainly into groups 1, 2, and 3. Each group comprised 14 specimens. The cross-section of the samples was cylindrical, square, and rectangular in groups 1, 2, and 3, respectively. Two specimens were tested within each group without any strengthening and functioned as the reference for other samples. Two identical samples were prepared for each specimen type^25,43^. Six of the other samples were strengthened using hemp confinement, whereas the other six were reinforced by utilizing cotton fibers. For each strengthening type, 1, 2, and 3 layers were employed to evaluate the effect of natural FRRP on the peak strength and strain (see Table 1). Each layer quantity was applied to two specimens to assess the consistency in results. A 3-part notation recognized each strengthened specimen. The first part referred to the shape (i.e., C for circular, S for square, and R for rectangular), the second part referred to the number of FRRP layers (i.e., 1, 2, or 3), and the third part denoted the nature of FRRP applied (i.e., H for hemp and C for cotton). For example, R-3H referred to the rectangular specimen strengthened with three layers of hemp FRRP.

**Table 1:** Summary of the experimental program.

ID	Groups	FRRP	FRRP Layers	Cross-Section
C-CON	1	–	–	Circular
C-1H		Hemp	1	Circular
C-2H		Hemp	2	Circular
C-3H		Hemp	3	Circular
C-1C		Cotton	1	Circular
C-2C		Cotton	2	Circular
C-3C		Cotton	3	Circular
S-CON	2	–	–	Square
S-1H		Hemp	1	Square
S-2H		Hemp	2	Square
S-3H		Hemp	3	Square
S-1C		Cotton	1	Square
S-2C		Cotton	2	Square
S-3C		Cotton	3	Square
R-CON	3	–	–	Rectangular
R-1H		Hemp	1	Rectangular
R-2H		Hemp	2	Rectangular
R-3H		Hemp	3	Rectangular
R-1C		Cotton	1	Rectangular
R-2C		Cotton	2	Rectangular
R-3C		Cotton	3	Rectangular

### Details of tested columns

**Figure 1:**
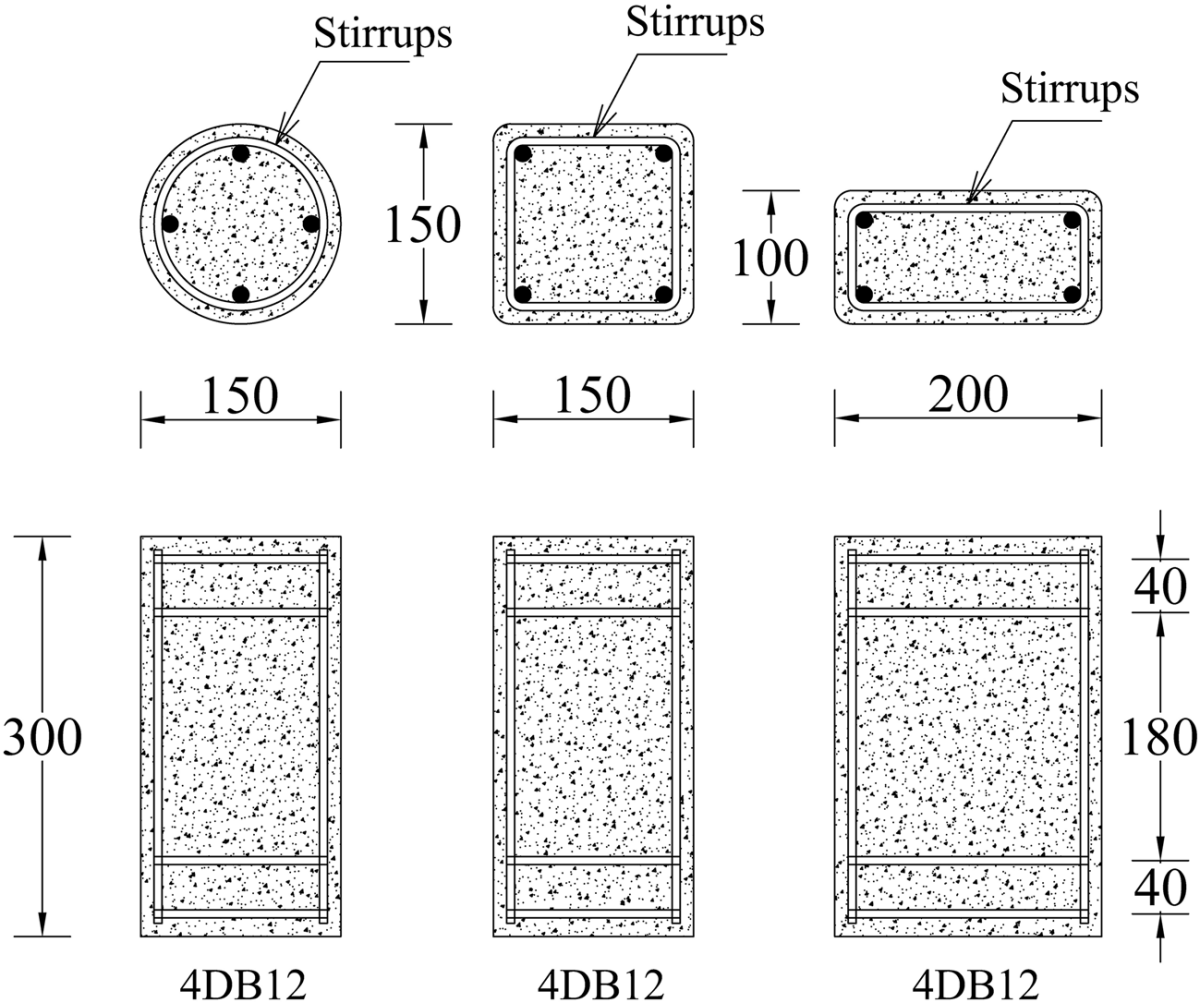
Structural details of test specimens (unit: mm).

Figure 1 summarizes the structural details of the specimens. The circular section was 150 mm in diameter, the square section had a side of 150 mm, and the rectangular section measured 150 mm $\times$ 100 mm. The height of the specimen of each section type was 300 mm. The reinforcement for the specimens consisted of four deformed bars with a 12-mm diameter as longitudinal reinforcement while four round bars with a 6-mm diameter were used as transverse reinforcement or stirrups. The spacing of transverse reinforcement was selected to provide slenderness ratio of 15 (L/D = 180/12 = 15 to ensure higher probability of buckling of longitudinal reinforcement. It is noteworthy that the same slenderness ratio has been adopted in earlier studies to account for the adverse effects of buckling and resulting localized stresses on the performance of external confinement and represent the commonly observed slenderness ratios in older structures^44^. Where L is length of bar between central stirrups and D is the dimeter of longitudinal reinforcement. Further, an additional stirrup was provided at each end to avoid crushing of the concrete as shown in Fig. 1. A minimum concrete cover of 15 mm was provided for all specimens.

### Material properties

**Figure 2:**
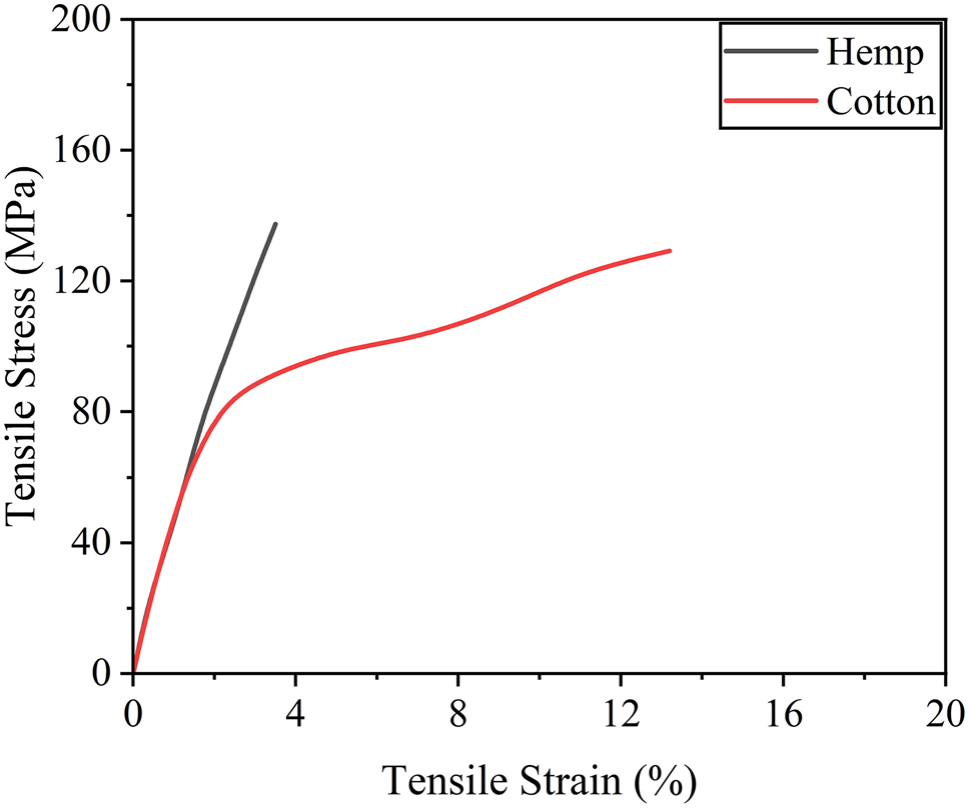
Tensile stress versus strain behavior of natural fiber composites.

Type-I ordinary Portland cement was utilized in the study, along with natural river sand as fine aggregate. The concrete mix proportions are presented in Table 2, with a target 28-day compressive strength of 20 MPa. The tested concrete strength for circular, square, and rectangular sections was 21.1 MPa, 20.7 MPa, and 27.2 MPa, respectively. Longitudinal reinforcement was comprised of deformed rebars with a diameter of 12 mm, while round rebars with a diameter of 6 mm were used for the transverse reinforcement. The tested yield strength of 12-mm and 6-mm diameter bars was 450 MPa and 340 MPa, respectively. Whereas the ultimate strength of 12-mm and 6-mm diameter bars was 620 MPa and 470 MPa, respectively. Locally available hemp and cotton fiber ropes and a two-part epoxy resin in 2:1 (epoxy: hardener) were used to prepare FRRP composites. The peak tensile capacity and the corresponding strain of the epoxy resin were 50 MPa and 2.5%, respectively. Natural fiber ropes were epoxy-impregnated and tensile tests were performed following^45,46^. The diameter of hemp and cotton fiber rope was 2.1 mm and 2.4 mm. The typical stress–strain curves of hemp and cotton FRP are shown in Fig. 2. Further properties of FRPs are given in Table 3.

**Table 2:** Concrete mix proportions.

Ingredient	Quantity (${\text{kg}}/{{\text{m}}}^{3})$
Cement	310
Sand	770
Aggregates	1100
Water	220

**Table 3:** Properties of FRPs.

Rope	Tensile strength (MPa)	Rupture strain (%)	Diameter (mm)
Hemp	137.4	3.5	2.1
Cotton	129.3	13.5	2.4

### Preparation of tested columns

**Figure 3:**
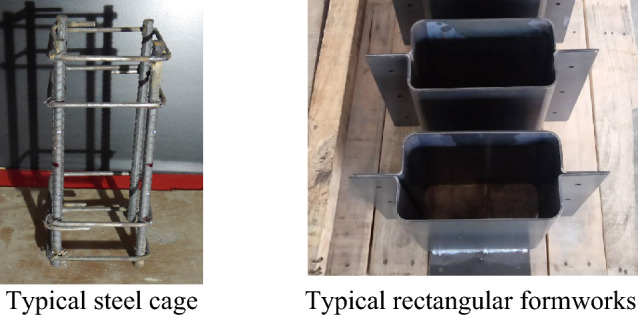
Typical preparation of test specimens.

Special steel molds were prepared for the specimens, as shown in Fig. 3. The steel cage was prepared outside the formwork for each specimen. This was practiced for the ease of preparation of the steel cage. After preparing steel cages, they were placed inside molds at the required positions. The inside surface of the steel molds was lubricated before pouring the concrete. Concrete was poured in three equal layers for each specimen, and a mechanical vibrator was used for the compaction of each layer. The steel molds were taken off the specimens after 24-h of concrete pouring, and the specimens were then subjected to a curing process for a duration of 28 days.

### Strengthening process

**Figure 4:**
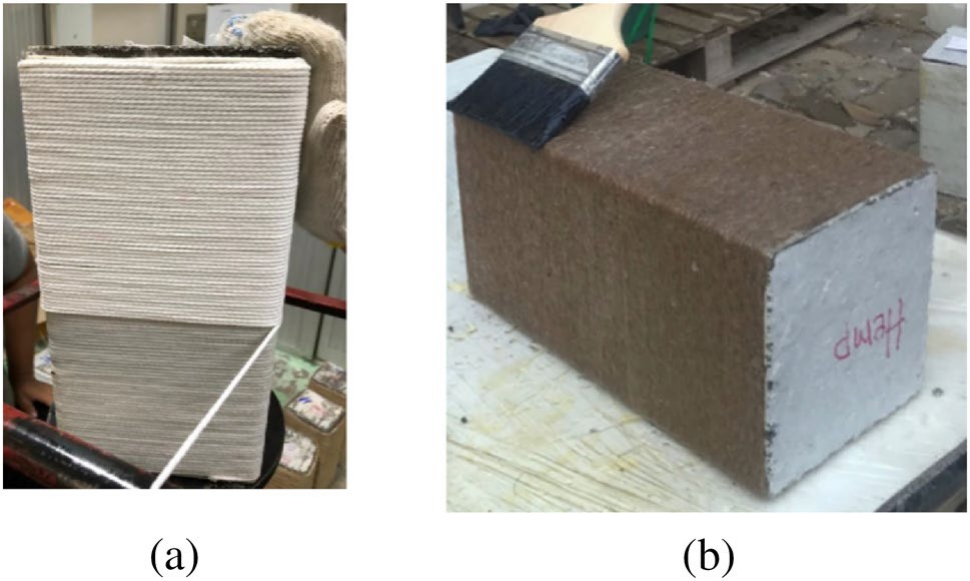
(**a**) A typical FRRP wrap in process and (**b**) epoxy impregnation of the wrapped FRRP layer.

Fiber ropes were applied to the specimens by hand. At first, the surface of specimens was impregnated with epoxy. Then, dry ropes were wrapped around the specimens in a vertical direction. The last end of rope was connected to the concrete using special glue to prevent any undesirable slip. After the complete wrapping of the specimen, the end of the rope was also fixed to the concrete. Special attention was paid to avoiding gaps between the successive wraps of ropes in the same layer. Once the dry wrap was completed, epoxy was put on the layer by means of a hand brush. An adequate amount of epoxy was used for a single layer to soak the ropes completely. In cases where specimens were strengthened with more than one layer of natural FRRP, a time gap of 12 h was provided before applying the subsequent FRRP layer. A similar process was also adopted for the application of subsequent FRRP layers. A typical strengthening process is illustrated in Fig. 4.

### Instrumentation and test setup

**Figure 5:**
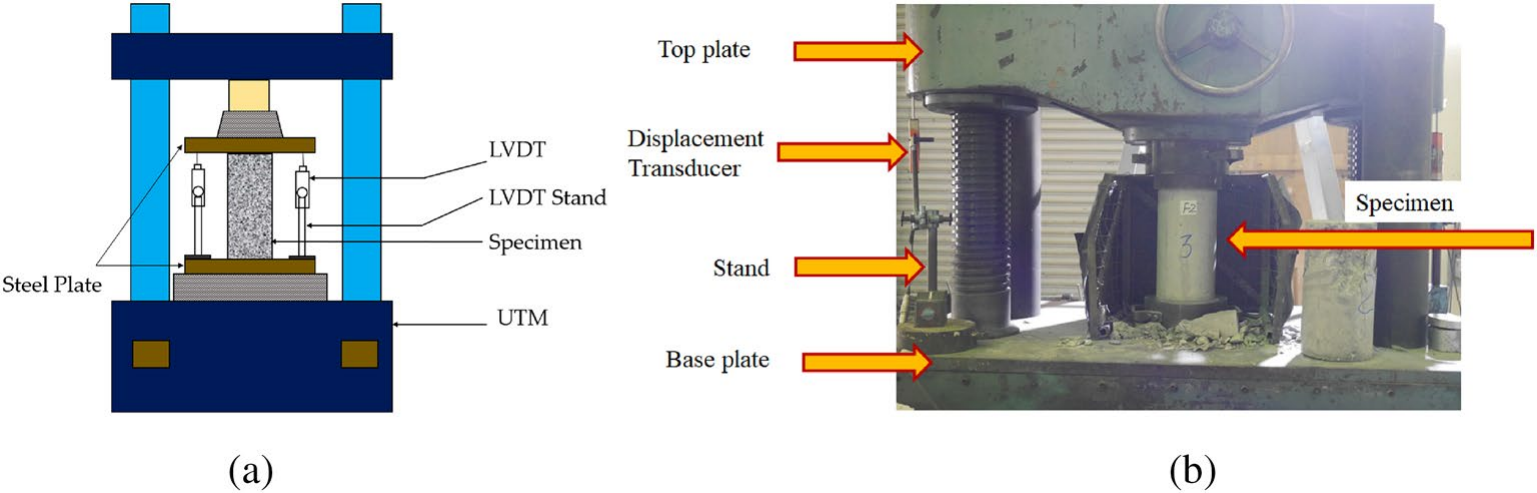
Typical compression test setup. (**a**) Schematic (**b**) Laboratory.

To assess the performance of each specimen, monotonic axial compression tests were conducted using a Universal Testing Machine with a capacity of 2000 kN, as illustrated in Fig. 5. The loading was applied in a load-controlled manner at a rate of 4 kN/s. Two displacement transducers were devoted to monitor the axial compression of the samples, with the tip of the transducers attached to the upper steel plate (as shown in Fig. 5). The intensity of the applied axial compression was monitored using a load cell that had a capacity of 1500 kN, which was positioned between the loading plate and the top of the specimen. All monitored data was recorded using a data logger.

## Experimental results

### Ultimate failure modes

**Figure 6:**
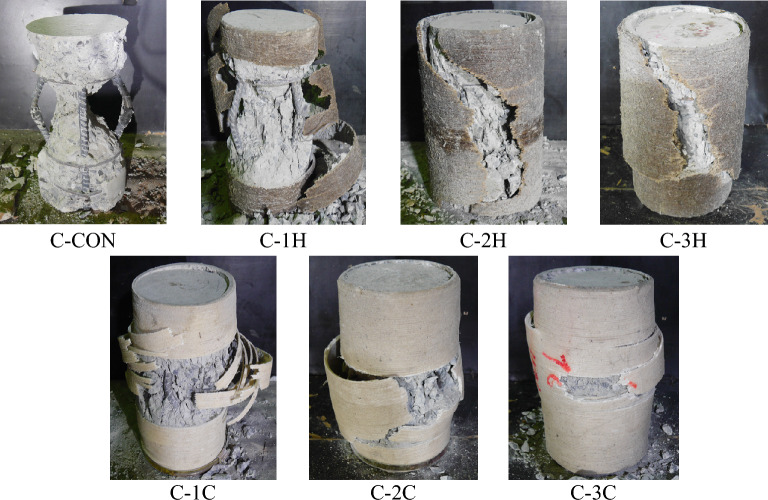
Failure modes of Group 1 samples.

The ultimate failure modes of Group 1 specimens are illustrated in Fig. 6. Group 1 specimens were cylindrical. As mentioned in Table 1, two specimens were tested for each strengthening type and reference. Ultimate failure modes discussed here represent the two samples for each specimen class in Table 1. The failure type of the reference Specimen C-CON was sudden and explosive. It accompanied the delamination of concrete cover resulting in the loss of confinement to the rebars. As a result, buckling of the rebars was observed with a further increase in the load leading to the crushing of the concrete core. The failure of specimens C-1H and C-1C were similar and exhibited the rupture of FRRP in the hoop direction. Specimens C-1H and C-1C were strengthened using a single FRRP layer that was proved insufficient to restrain the buckling of longitudinal rebars, as shown in Fig. 6. Failure of the specimens C-2H and C-2C included the rupture of FRRP layers. However, for the hemp confined Specimen C-2H, the rupture of FRRP progressed along the full height of the specimen, whereas the rupture was contained in the middle for the cotton FRRP confined specimen. Further, a slight buckling of longitudinal rebars was observed for Specimen C-2C. For three FRRP-layer confined circular specimens, C-3H and C-3C, the failure pattern was analogous to the two FRRP-layer confined specimens. However, the magnitude of the core concrete crushing was reduced, and no buckling of the longitudinal rebars was experienced, highlighting that a 3-layer FRRP confining system provided adequate confinement. The slenderness ratio of 15 was adopted to ensure buckling of longitudinal reinforcement and to evaluate the efficacy of natural fiber confinement in preventing buckling. The phenomenon of buckling of longitudinal reinforcement was observed predominantly in control specimen. Slight traces of buckling were observed for single layer of cotton and hemp confinements, whereas buckling of steel bars was reduced when 2 or 3 layers of natural fibers were used as compared to control and single layer of cotton and hemp confinements.

**Figure 7:**
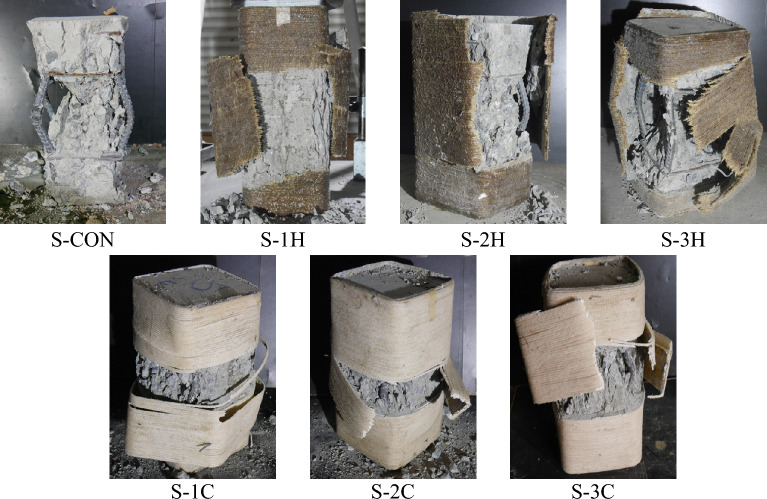
Failure types of Group 2 specimens.

Figure 7 illustrates the failure modes of Group 2 samples. The control Specimen S-CON exhibited a brittle crushing of the core and cover concrete along with the buckling of longitudinal rebars. The extent of concrete crushing and the magnitude of brittleness was significantly reduced in the case of strengthened specimens. As seen in Fig. 7, the failure of all strengthened samples in Group 2 accompanied the failure of FRRP in the hoop direction. More importantly, the location of the rupture was observed near the sharp corners irrespective of the quantity and type of natural FRRP wraps. This occurred despite the provision of a 13 mm corner radius in rectilinear samples. ACI SPEC-440.12-22^47^ advises providing at least a 13 mm corner radius in rectilinear sections to avoid stress concentrations in external FRP systems. The provisions of ACI SPEC-440.12-22 are based on the experimental findings of synthetic FRP strengthened systems. Therefore, the same minimum requirement of the corner radius may not be true for natural FRRP strengthened rectilinear sections. Similar to circular specimens, severe buckling of longitudinal reinforcement, as observed in Specimen S-CON, was substantially reduced, especially for 2 or 3 layers of confinement.

**Figure 8:**
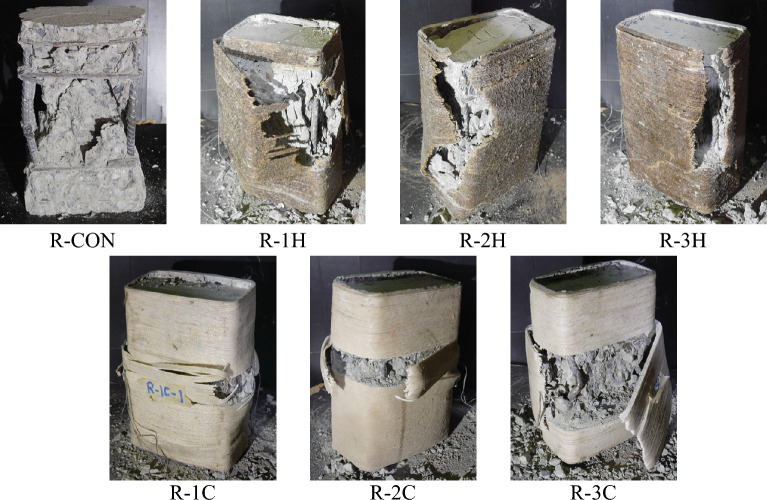
Failure modes of Group 3 samples.

The Group 3 specimens underwent axial compression testing, and their ultimate failure modes are depicted in Fig. 8. The control sample, R-CON, exhibited failure due to longitudinal rebar buckling and core concrete crushing in a brittle manner. On the other hand, strengthened specimens exhibited FRRP rupture in the hoop direction near the corners of the specimens. Interestingly, the rupture of FRRP occurred near the corners, irrespective of the number of FRRP layers. This suggests that the provision of a 13 mm corner radius was inadequate in inhibiting stress concentrations near corners in rectangular specimens. As a result, the efficiency of FRRP layers was compromised, leading to buckling of longitudinal rebars even for specimens with 3-layer FRRP confinement. An example of this is seen in the failure mode of Specimen R-3C in Fig. 8.

### Stress versus strain curves

**Figure 9:**
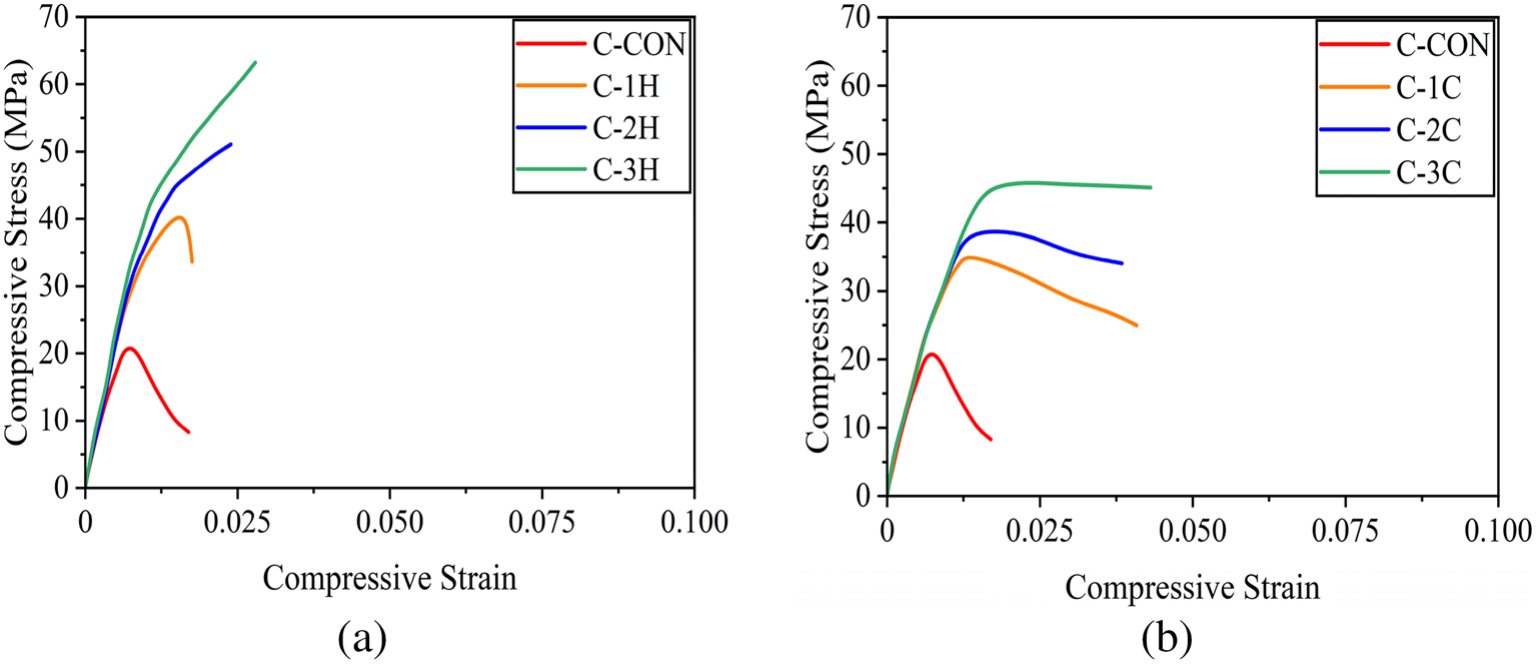
Compressive stress–strain response of circular sections (**a**) hemp FRRP and (**b**) cotton FRRP.

It is noteworthy that some studies have presented compressive stresses of reinforced concrete sections exclusively for concrete by subtracting the contribution of longitudinal reinforcement^37^. However, the stresses computed in the present study correspond to the average stress experienced by the full section, i.e., without excluding the contribution from longitudinal reinforcement^49,52^. Figure 9a presents the compressive stress–strain response of circular sections confined with the hemp confinement. The control specimen C-CON exhibited a steep initial branch till the maximum capacity followed by an abrupt loss in its strength demonstrating a brittle failure. The compressive strength increased with the number of hemp FRRP layers. For one- and two-layer hemp confinement, the initial steep branch was followed by a milder branch till the rupture of hemp FRRP. At this point, specimens dropped their capacity. For the three-layer hemp confinement, a bilinear stress–strain response was observed till high compressive strain values. The application of cotton FRRP also improved the compressive response of circular reinforced concrete specimens (Fig. 9b). However, a distinct shape of the compressive stress–strain behavior was observed. For one and two-layer cotton confinement, the initial steep branch was followed by a descending branch, whereas a stable second branch was observed in the case of three-layer cotton confinement. Nonetheless, both confinement types greatly improved the compressive ductility of circular reinforced concrete sections.

**Figure 10:**
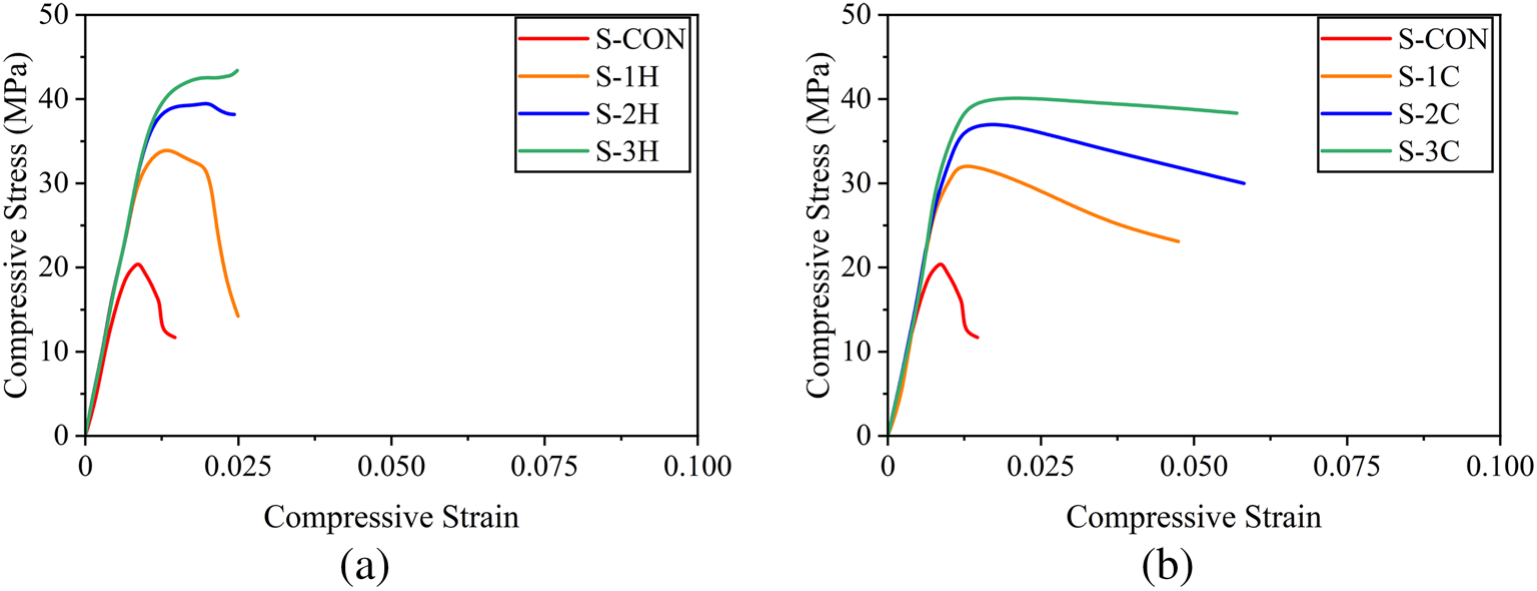
Compressive stress–strain response of square sections (**a**) hemp FRRP and (**b**) cotton FRRP.

The measured stress–strain behavior of square specimens strengthened with the hemp confinement is shown in Fig. 10a. A descending branch in the case of the two-layer hemp confinement was encountered. This is contrary to the same confinement on circular sections where an ascending second branch was observed. This suggests that the efficiency of hemp FRRP was reduced on square sections. The axial capacity of square sections did not increase beyond the initial steep branch. However, hemp FRRP was able to prevent compressive failure till large strain values. In the case of cotton confinement on square sections (see Fig. 10b), a bilinear compressive stress–strain response was observed with a descending second branch irrespective of the number of layers.

**Figure 11:**
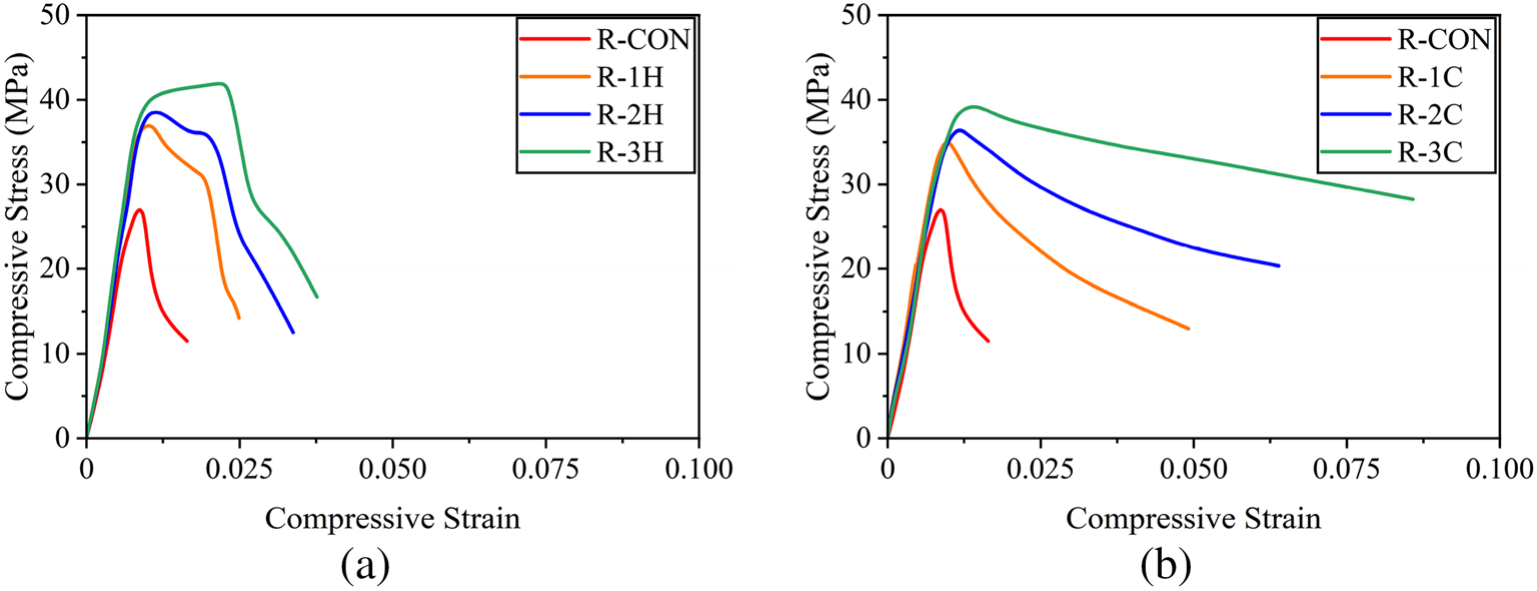
Compressive stress–strain response of rectangular sections (**a**) hemp FRRP and (**b**) cotton FRRP.

The performance of hemp and cotton confinement further deteriorated on rectangular sections as shown in Fig. 11. The length of the second stable branch in the case of hemp confinement was reduced, whereas the steepness of the second branch in the case of cotton confinement increased.

### Compressive strength and corresponding strain

In the present study, the term “compressive strength” referred to the maximum stress observed in stress–strain curve, whereas the “ultimate stress” was defined as the stress observed at the failure. The key objective of the present study was to assess the role of natural FRRP in enhancing the axial compressive performance of the concrete. Experimental results in terms of the compressive strength and the corresponding strain are summarized in Table 4. For circular columns, an increase of 92%, 142%, and 200% in the compressive strength was detected for 1, 2, and 3 wraps of hemp FRRP confinement, respectively, whereas the corresponding increase in the case of cotton FRRP was 68%, 84%, and 117%, respectively. It is desired from the external confinement to impart axial ductility to the concrete, which is normally assessed by the strain at failure. The last column of Table 4 presents the increase in axial compressive strain at the ultimate failure. For circular columns, 1, 2, and 3 layers of hemp FRRP enhanced the peak strain by 91%, 216%, and 270%, respectively. The corresponding increase for the case of cotton confinement was 76%, 134%, and 233%, respectively. For square columns in Group 2, an increase of 66%, 91%, and 110% in the compressive strength was detected for 1, 2, and 3 wraps of hemp FRRP confinement, respectively, whereas the corresponding increase in the case of cotton FRRP was 58%, 88%, and 95%, respectively. The peak strain for 1, 2, and 3 layers of hemp FRRP increased by 49%, 132%, and 186%, respectively, whereas the corresponding increase in the case of cotton FRRP was 38%, 103%, and 144%, respectively. Similarly, enhancement in compressive strength and peak strain of up to 47% and 144% was observed for 3-layer hemp FRRP, whereas the corresponding increase for a 3-layer cotton FRRP was up to 46% and 58%, respectively.

**Table 4:** Summary of compressive strength and strain.

ID	Compressive strength (MPa)	Improvement in stress (%)	Strain at compressive strength	Improvement in strain (%)
C-CON	21.1	–	0.0076	–
C-1H	40.5	92	0.0145	91
C-2H	51.1	142	0.0239	216
C-3H	63.3	200	0.0279	270
C-1C	35.5	68	0.0133	76
C-2C	38.9	84	0.0177	134
C-3C	45.8	117	0.0252	233
S-CON	20.7	–	0.0087	–
S-1H	34.4	66	0.0129	49
S-2H	39.5	91	0.0202	132
S-3H	43.4	110	0.0248	186
S-1C	32.7	58	0.0120	38
S-2C	37.3	80	0.0176	103
S-3C	40.3	95	0.0212	144
R-CON	27.2	–	0.0091	–
R-1H	37.3	37	0.0097	7
R-2H	38.5	41	0.0133	47
R-3H	40.0	47	0.0221	144
R-1C	37.3	35	0.0108	10
R-2C	38.7	35	0.0122	29
R-3C	42.0	46	0.0221	58

### Effect of shape

**Figure 12:**
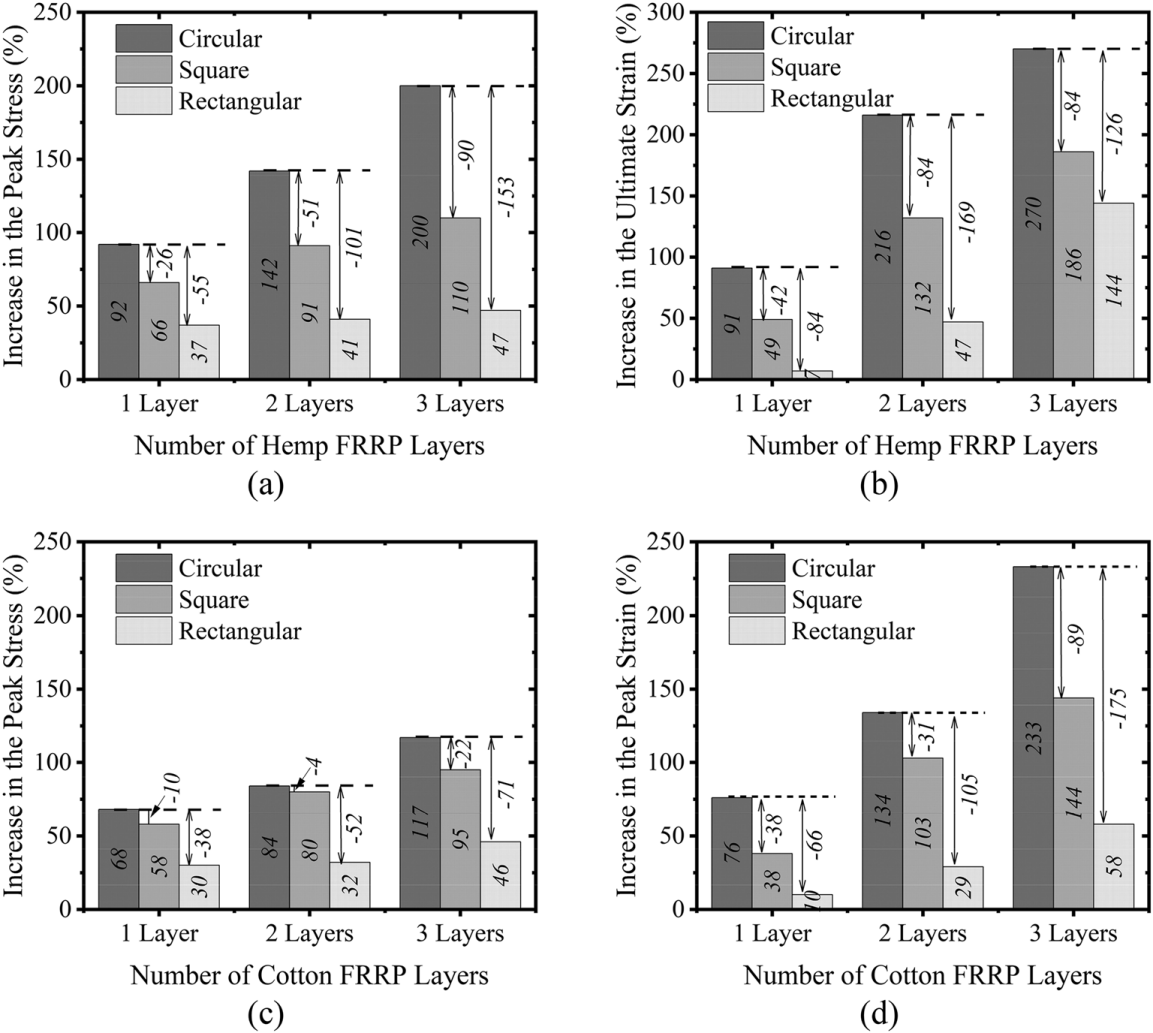
Increase in (**a**) peak stress due to hemp FRRP (**b**) peak strain due to hemp FRRP (**c**) peak stress due to cotton FRRP and (**d**) peak strain due to cotton FRRP.

The influence of the cross-section shape on enhancement of compressive strength and corresponding strain by hemp FRRP confinement is presented in Fig. 12a,b. It is apparent in Fig. 12a that the increase in the compressive strength was dependent on the cross-sectional shape of the column. The highest gain was observed in the case of circular columns. This was expected as the circular columns are uniformly confined, and the maximum efficiency of the external wraps can be utilized. Though a corner radius of 13 mm was incorporated in rectilinear sections before applying FRRP confinement, the corner radius of 13 mm was inadequate to alleviate the concentration of stresses fully in rectilinear sections. The results were worse in rectangular sections as compared to square sections. A similar observation can be made in Fig. 12b, where the highest enhancement in the peak strain due to hemp FRRP confinement was observed by circular sections followed by square and rectangular sections, respectively.

Further, the deviation between the improvement in the compressive strength and corresponding strain improved with the quantity of hemp FRRP wraps were incremented. For instance, one wrap of hemp FRRP enhanced the compressive strength of circular, square, and rectangular sections by 92%, 66%, and 37%, respectively. This corresponds to the reduced efficiency of hemp FRRP confinement on square and rectangular by 26% and 55%, respectively (see Fig. 12a). Two wraps of hemp FRRP increased the compressive strength by 142%, 91%, and 41% in circular, square, and rectangular sections, respectively. Compared to the 1-layer hemp confinement, the difference in the gain (%) of compressive strength by square and rectangular sections for two layers of hemp FRRP confinement increased to 51% and 101%, respectively. A similar observation can also be made for 3-layer hemp FRRP strengthening in Fig. 12a. Figure 12c,d represent the increase in the compressive strength and peak strain ascribed to cotton FRRP confinement. Here, the maximum efficiency of the cotton FRRP confinement was observed for circular sections again, followed by square and rectangular sections, respectively. In general, the distinction in the enhancement of the compressive strength and peak strain by circular and rectilinear sections increased as cotton FRRP wraps increased.

### Strengthening materials

**Figure 13:**
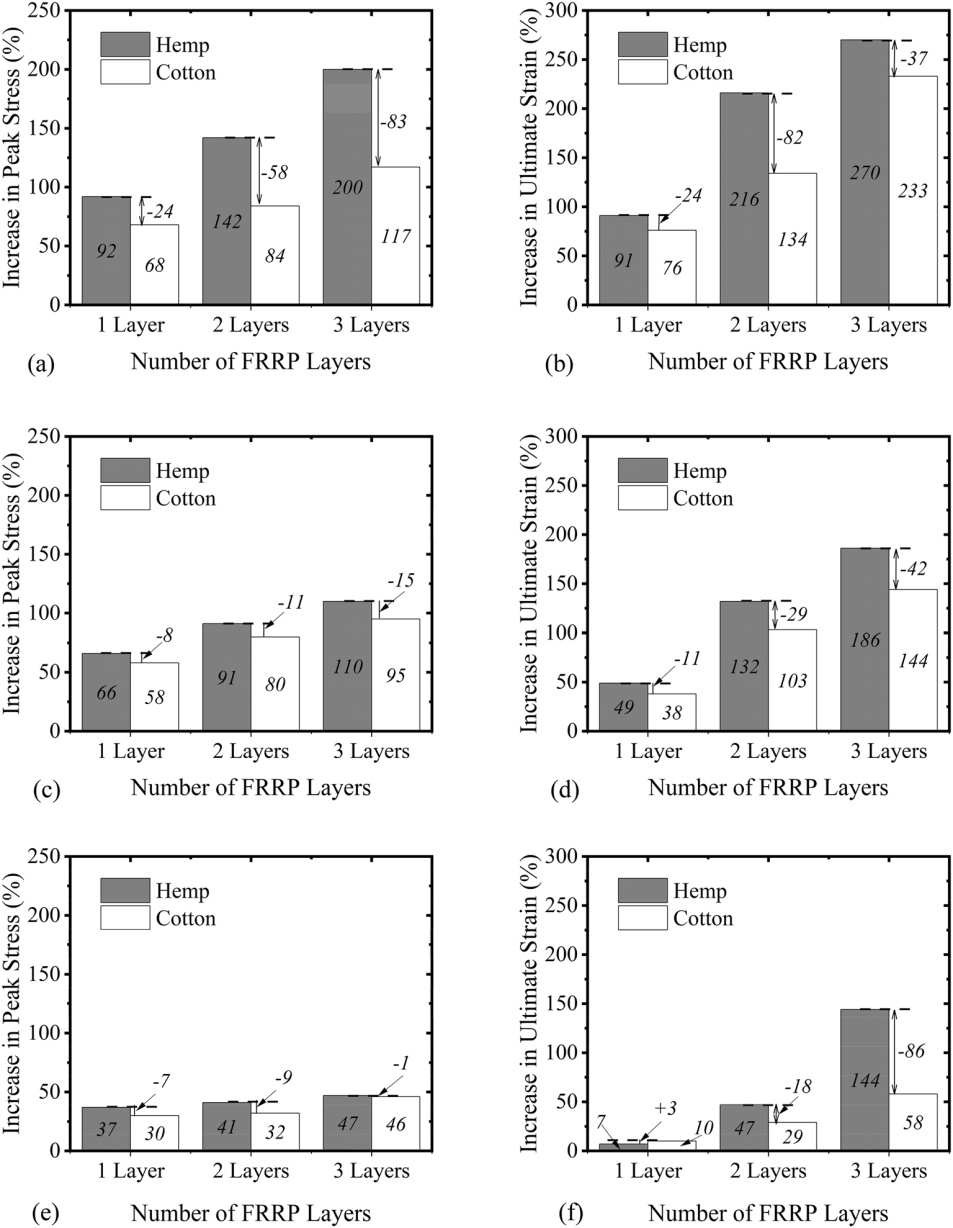
Comparison of the enhancement in mechanical properties of concrete by hemp and cotton FRRP layers. (**a**) Increase in the peak stress of circular sections (**b**) Increase in the peak strain of circular sections (**c**)Increase in the peak stress of square sections (**d**)Increase in the peak strain of square sections (**e**)Increase in the peak stress of rectangular sections (**f**)Increase in the peak strain of rectangular sections.

Figure 13a,b present the comparison for the case of circular specimens. For hemp confinement on circular specimens, the enhancement in the compressive strength by 1, 2, and three layers was 24%, 58%, and 83% higher than the corresponding increase due to cotton FRP confinement. Similarly, 1, 2, and 3 layers of hemp FRRP increased the peak strain by 91%, 216%, and 270%, respectively, which was found to be 24%, 82%, and 37% higher for cotton confined circular specimens. This indicates that for similar characteristics of the underlying reinforced concrete, the enhancement in the compressive strength and peak strain would be higher for hemp FRRP than cotton FRRP for a similar number of layers. In other words, to achieve a similar enhancement in the compressive strength and compressive ductility in circular sections, a larger number of cotton FRRP layers would be required as compared to hemp FRRP layers. Figure 13c,d illustrate the comparison in the compressive strength and strain of the square sections due to hemp and cotton FRRP confinement. A similar observation as that in the case of circular sections can be made. For a similar number of layers, the enhancement in the compressive strength and strain was greater for specimens strengthened with hemp confinement. But the deviation in enhancement in compressive strength and corresponding strain ascribed to hemp and cotton strengthening was smaller as compared to that in circular sections. A similar comparison for rectangular sections is presented in Fig. 13e,f. Interestingly, the effect of hemp and cotton confinement on the compressive strength and corresponding strain were quite similar for the same number of FRRP layers. The only noticeable difference was found in the peak strain gain for 3-layer hemp and cotton strengthening, as shown in Fig. 13f. In general, hemp FRRP confinement has shown a better potential for the enhancement in the compressive strength and corresponding strain than cotton FRRP confinement.

## Analytical investigations

In recent years, several analytical models have been proposed to predict the compressive strength and ultimate compressive strain of the concrete that is confined by FRP jackets^37,39,49–59^. It is known that synthetic FRP jackets provide confinement through their in-plain stiffness mainly^60^. Since the considered hemp and cotton FRRP confinements comprised uniaxial characteristics only, it is justified to use existing FRP confined concrete stress–strain models to natural FRRP confined concrete and evaluate their accuracy.

### Prediction of the compressive strength and ultimate compressive strain for circular sections

The ultimate strain was defined differently for specimens with ascending and descending post-peak branches. For specimens with an ascending post-peak branch, the ultimate strain was defined to be the maximum strain observed till failure^37,49^. Whereas the ultimate strain was defined to be the strain corresponding to a 20% drop in peak capacity when a descending post-peak branch was observed^61^. The models of Pellegrino and Modena^49^, Wei et al.^51^, Hamid et al.^50^, Chastre and Silva^39^, Lee et al.^52^, Issa et al.^55^, Ilki and Kumbasar^56^, Ilki et al.^37^, Huang et al.^57^, and Harajli^58^ were used to forecast the compressive strength, and the ultimate strain of the hemp and cotton FRRP confined concrete with circular sections. These models were selected as these models consider the interaction between the external FRP confinement and the internal transverse reinforcement in improving the overall axial compressive performance of the concrete. Table 5 presents the average value of the ratios of experimental to predicted values of compressive strength (this ratio was referred to as “AVG”) and ultimate strains for the considered analytical models. The standard deviations (SD) are also listed next to each (AVG) value. The models of Lee et al.^52^ and Ilki et al.^37^ resulted in AVG values of 0.97 and 0.96, respectively for the compressive strength. However, high SD values of about 14% were associated with them, indicating that the predicted compressive strength by models of Lee et al.^52^ and Ilki et al.^37^ were inconsistent despite their close-to-one AVG values. For ultimate strains, the closest-to-one AVG value was given by the expression of Issa et al.^55^ but it accompanied an SD value of about 20%.

**Table 5:** Statistical assessment of existing stress–strain models for circular columns.

Models for circular sections	Columns in Group 1			
	Peak stress		Ultimate strain	
	AVG	SD	AVG	SD
Pellegrino and Modena^49^	1.09	0.14	0.93	0.11
Wei et al.^51^	0.74	0.14	–	–
Hamid et al.^50^	2.64	0.38	–	–
Chastre and Silva^39^	0.74	0.13	0.33	0.08
Lee et al.^52^	0.97	0.13	1.61	0.57
Issa et al.^55^	1.40	0.20	0.97	0.20
Ilki and Kumbasar^56^	0.93	0.14	0.89	0.08
Ilki et al.^37^	0.96	0.14	0.74	0.11
Huang et al.^57^	1.08	0.17	0.67	0.06
Harajli^58^	0.54	0.06	0.30	0.06

AVG is the average value of the ratios of experimental to predicted values of compressive strength.

### Prediction of the compressive strength and ultimate strain for rectilinear sections

For rectilinear sections, the models of Pellegrino and Modena^49^, Ilki et al.^37^, Harajli^58^, and Eid and Paultre^59^ were considered. Table 6 presents the average value of the ratios of experimental to predicted values of compressive strength (AVG) and ultimate strains for the considered analytical models. The standard deviations (SD) are also listed next to each (AVG) value. The model of Ilki et al.^37^ yielded an AVG value of 1.04 for the peak stress at the corresponding SD value of about 8%, whereas the AVG value of the peak strain was 0.55 with an SD value of about 35%. None of the remaining models were able to predict the compressive strength and the ultimate strain with satisfactory accurateness. Therefore, it can be inferred that existing models of FRP confined concrete are unable to estimate the mechanical properties of natural FRRP confined concrete.

**Table 6:** Statistical assessment of existing stress–strain models for rectilinear columns.

Models for rectilinear sections	Columns in Groups 2 and 3			
	Peak stress		Ultimate strain	
	AVG	SD	AVG	SD
Pellegrino and Modena^49^	0.82	0.18	0.71	0.15
Ilki et al.^37^	1.04	0.08	0.55	0.35
Harajli^58^	0.84	0.18	0.48	0.20
Eid and Paultre^59^	0.96	0.05	1.78	0.60

AVG is the average value of the ratios of experimental to predicted values of compressive strain.

### Proposed analytical models

#### Idealized curves for confined concrete

**Figure 14:**
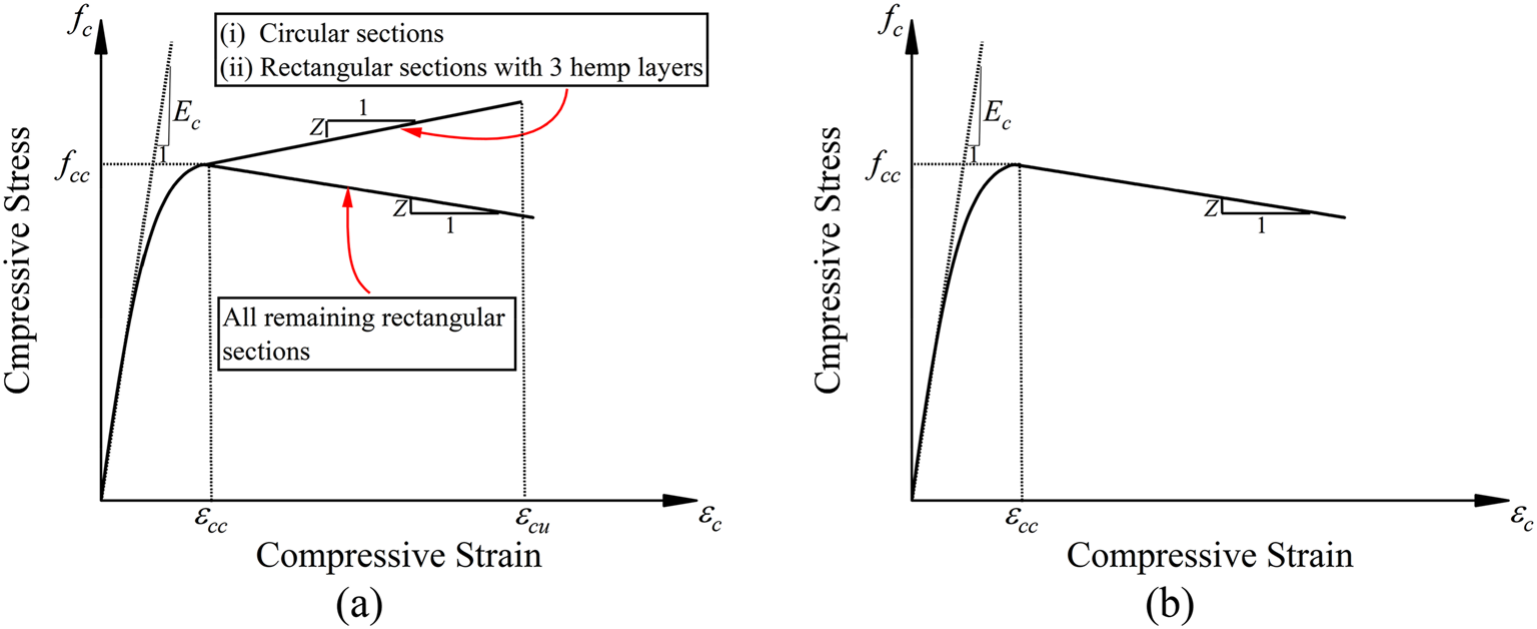
Idealized compressive stress versus strain behavior for (**a**) hemp confinement and (**b**) cotton confinement.

It was observed from experimental stress–strain curves of confined concrete that the post-peak behavior of hemp and cotton confined concrete was different. For rectilinear sections, the hemp confinement resulted in both ascending and descending post-peak branches, depending upon the level of confinement. It is important to note that this observation must be studied parallel to the confinement type and quantity adopted in the present study. Thus, the idealized stress–strain curves for both types of confinement are shown in Fig. 14. It is apparent that the initial modulus ${E}_{c}$, the post-peak modulus $Z$, the compressive strength ${f}_{cc}$, the peak compressive strain ${\epsilon }_{cc}$ were required to trace the compressive stress–strain curves. An additional parameter in the form of the ultimate strain was required when an ascending post-peak branch was encountered (see Fig. 14a). The stress–strain behavior till the peak point was observed to be parabolic. It was assumed that the initial stress versus strain curves of recycled aggregate concrete would follow a parabolic shape until the compressive strength is achieved. To achieve this, the expressions proposed by Popovics^62^, subsequently revised by Mander et al.^63^, were implemented. The stress ${f}_{c}$ at any general strain $\epsilon$ is given as:

1 $${f}_{c}={f}_{cc}\frac{x\times r}{r-1+{x}^{r}}$$

2 $$x=\frac{\epsilon }{{\epsilon }_{cc}}$$

3 $$r=\frac{{E}_{c}}{\left({E}_{c}-\frac{{f}_{cc}}{{\epsilon }_{cc}}\right)}$$

where distinguished expressions for the elastic modulus ${E}_{c}$, ${f}_{cc}{\prime}$, and ${\epsilon }_{cc}$ will be presented. After the compressive strength is achieved, expressions for the degradation modulus $Z$ will be proposed. The following sections demonstrate the regression results performed to predict different characteristic points on the curve. It is important to note that the post-peak behavior of hemp confined rectilinear sections was not consistent, i.e., both the ascending and descending branches were encountered. Therefore, the compressive stress–strain behavior of hemp confined rectangular sections was not modeled. Thus, it could be inferred that further studies are required to better understand the second component (i.e., behavior post compressive strength) of compressive stress versus strain curves of rectilinear sections strengthened with hemp confinement.

#### Elastic modulus ${E}_{c}$

The elastic modulus of all confined specimens did not show significant variation. Therefore, a constant value of ${E}_{c}$ was proposed, as given:

4 $${E}_{c}=830\times \sqrt{{f}_{co}}$$

where ${f}_{co}$ is the compressive strength of unstrengthen concrete in MPa.

#### Adopted methodology for concrete modeling

In existing approaches^37,39,49,51,52,57,58^, the first step towards formulations for mechanical properties of confined concrete is to estimate the confinement pressure that is exerted by the external wraps. The confining pressure in the case of internal steel ${f}_{ls}$ and external wraps ${f}_{lf}$ must be computed individually to account for their interaction. Thus, the total confining pressure is the sum of the individual confining pressures by FRRP and the internal reinforcement. The details of the confining pressure calculation are provided in the Appendix

#### Model proposal for circular sections

##### *Compressive stress *${f}_{cc}$* and strain*${\epsilon }_{cc}$

**Figure 15:**
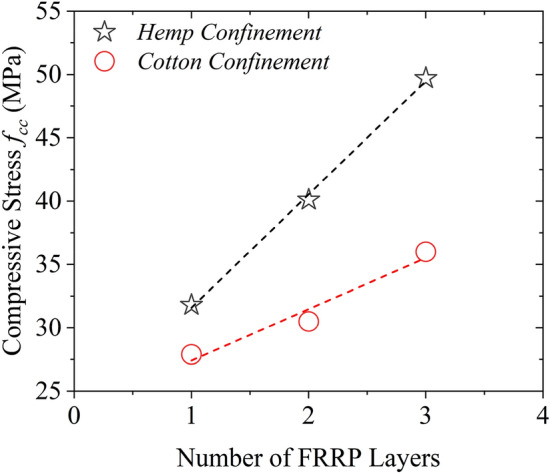
Hemp versus cotton confinement to improve the compressive stress ${f}_{cc}$.

Given that the confinement on circular and rectilinear sections perform differently, the expressions for the compressive stress ${f}_{cc}$ and strain ${\epsilon }_{cc}$ of hemp and cotton FRRP confined concrete were proposed separately for circular and rectangular sections. The form of equations used to propose the compressive strength and the peak strain is inspired by the formulations of Pellegrino and Modena^49^. The compressive stress ${f}_{cc}$ and strain ${\epsilon }_{cc}$ are defined in Eqs. (5) and (6), respectively.

5 $$\frac{{f}_{cc}}{{f}_{co}}=1+{k}_{A}\left(\frac{{f}_{u}}{{f}_{co}}\right)$$

6 $$\frac{{\epsilon }_{cc}}{{\epsilon }_{co}}=1+{k}_{B}\left(\frac{{f}_{u}}{{f}_{co}}\right)$$

where ${f}_{u}$ is defined in Appendix. The parameters ${k}_{A}$ and ${k}_{B}$ are defined in Eqs. (7) and (8), respectively.

7 $${k}_{A}=A{\left(\frac{{f}_{u}}{{f}_{co}}\right)}^{-\alpha }$$

8 $${k}_{B}=B{\left(\frac{{f}_{u}}{{f}_{co}}\right)}^{-\beta }$$

where $A$, $B$, $\alpha$, and $\beta$ are the constants of regression. These constants were regressed separately for hemp and cotton confinement. The Gauss–Newton algorithm was used to perform nonlinear regression. This is because the improvement in the mechanical properties of the concrete was different for both these confinements. For instance, for the same number of FRRP layers, the increase in the compressive stress ${f}_{cc}$ was higher for hemp confinement, as shown in Fig. 15. As discussed in earlier sections, the efficiency of the hemp FRRP was superior to that of the cotton FRRP for the same number of layers. A nonlinear regression analysis was performed to estimate the constants in Eqs. (7) and (8). Table 7 provides the estimated regression constants $A,\alpha , B,$ and $\beta$ for hemp and cotton confinements.

**Table 7:** Regression constants for circular sections.

Constant	Hemp confinement	Cotton confinement
$A$	1.57	1.49
$\alpha$	0.49	0.47
$B$	6.16	16.32
$\beta$	0.75	0.28

**Figure 16:**
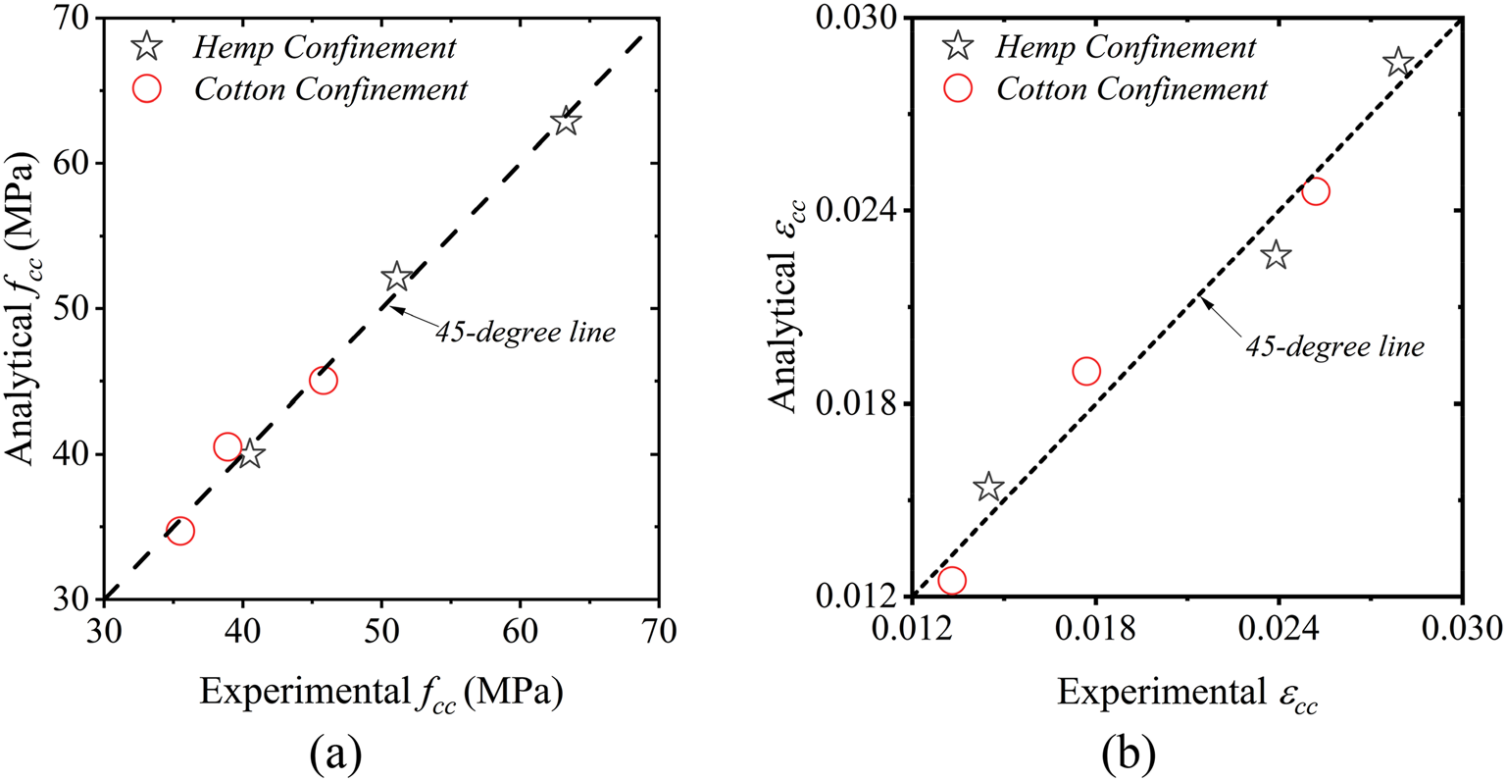
Comparison between analytical and experimental results for the circular confined concrete (**a**) compressive stress ${f}_{cc}$ and (**b**) strain ${\epsilon }_{cc}$.

Figure 16 provides the comparison between experimental and analytically predicted compressive stresses ${f}_{cc}$ and strains ${\epsilon }_{cc}$ of the confined concrete with circular sections. The prediction was carried out using the constants presented in Table 7. It is evident that a good agreement exists between the analytical and experimental compressive strength and peak strains.

##### *Post-peak modulus*$Z$

It was observed that ascending and descending post-peak branches were observed for concrete confined with hemp and cotton ropes, respectively. Thus, the post-peak modulus $Z$ needed to be differentiated in terms of the confinement type. For this purpose, different regression analysis was performed on the post-peak behavior of hemp and cotton confined concrete.

The variation in the post-peak modulus as a function of the level of external confinement are presented in Table 8. Notably, the modulus increased as the level of hemp confinement increased. Similarly, the modulus decreased as the level of cotton confinement decreased. Equations (9) and (10) were proposed to calculate the post-peak modulus of concrete having circular sections confined with hemp and cotton ropes, respectively. It is to be noted that Eqs. (9) and (10) resulted in the coefficient of determination of 0.77 and 0.86, respectively.

**Table 8:** Comparison of post-peak modulus for hemp and cotton confinement on circular sections.

Number of layers	Post-peak modulus (MPa)	
	Hemp	Cotton
1	765	− 381
2	801	− 236
3	1206	− 31

9 $$Z=289.51{\left(1+{f}_{u}\right)}^{0.54}$$

10 $$Z=-4550.61{\left(1+{f}_{u}\right)}^{-1.46}$$

##### *The ultimate strain*${\epsilon }_{u}$

It was mentioned that an additional parameter in the form of ultimate strain ${\epsilon }_{u}$ was needed to track the stress versus strain behavior of circular sections strengthened with hemp confinement. Similar to the peak strain, an equation for the ultimate strain of circular sections strengthened with hemp FRRP was proposed by performing regression analysis. Thus, the following equation was found adequate to predict ${\epsilon }_{u}$, with a coefficient of determination of 0.99.

11 $$\frac{{\epsilon }_{u}}{{\epsilon }_{co}}=1+17.74{\left(\frac{{f}_{u}}{{f}_{co}}\right)}^{0.52}$$

##### Experimental and predicted stress–strain responses

**Figure 17:**
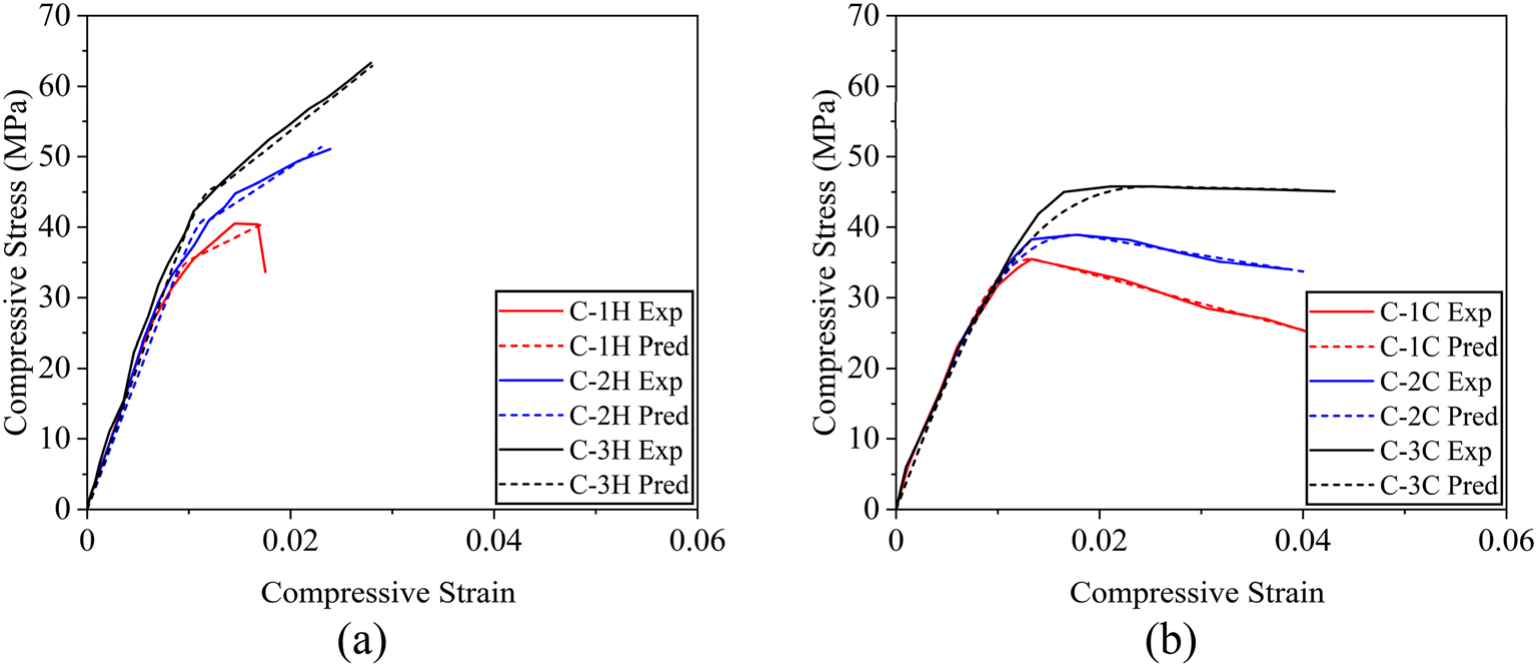
Stress–strain curves of circular sections for (**a**) hemp confinement and (**b**) cotton confinement.

The predicted stress versus strain curves for hemp confinement demonstrated a slight deviation in terms of the initial stiffness (Fig. 17). However, the post-peak behavior, the compressive strength, and the ultimate strain were well captured by the proposed scheme. Contrary to the hemp confinement, the predicted stress–strain curves did not only match the post-peak behavior and compressive strength, but it also traced the initial stiffness with reasonable accuracy.

#### Model proposal for rectilinear sections

##### Compressive stress ${{\varvec{f}}}_{{\varvec{c}}{\varvec{c}}}$ and strain ${{\varvec{\epsilon}}}_{{\varvec{c}}{\varvec{c}}}$

**Figure 18:**
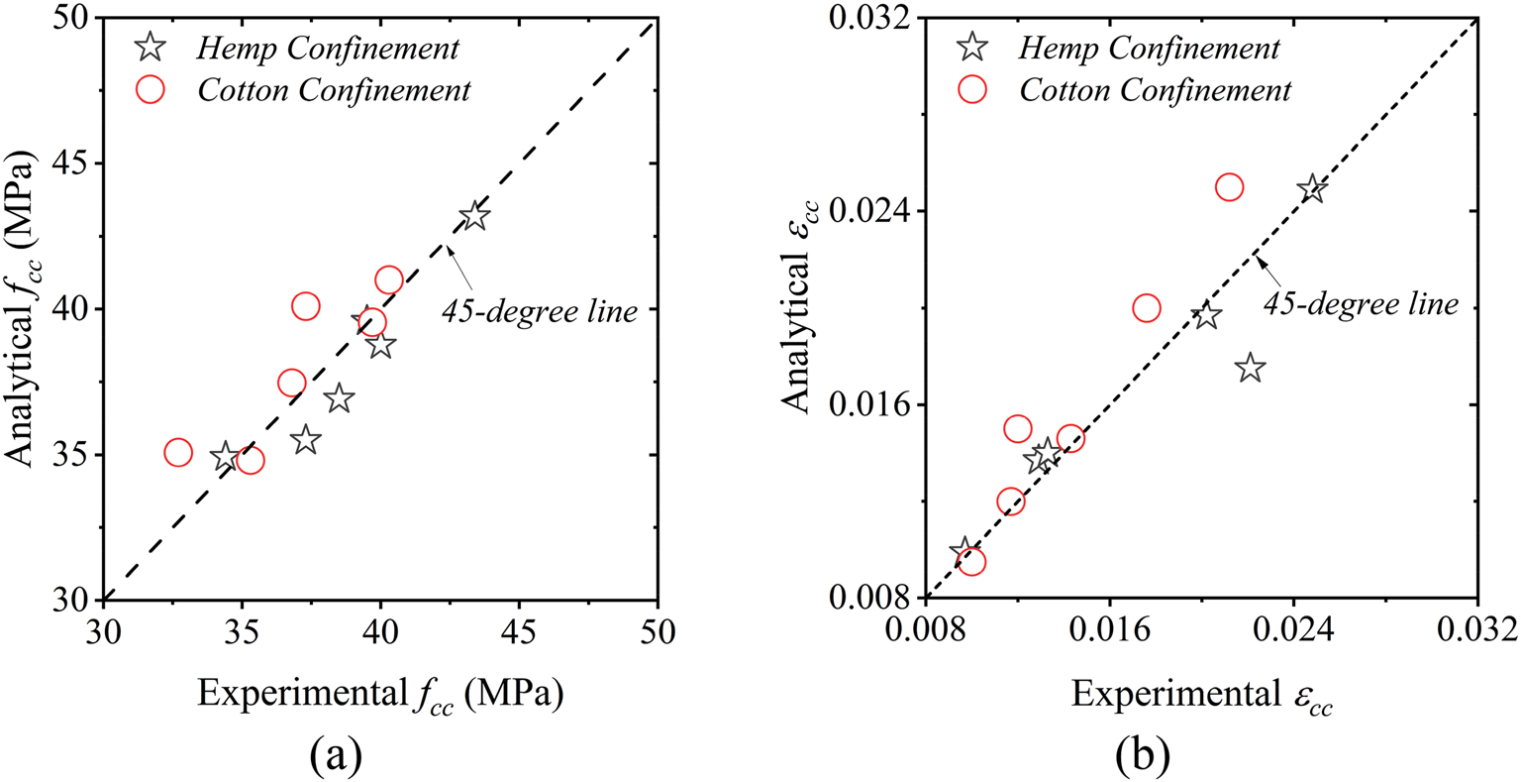
Comparison between analytical and experimental results for the confined rectangular concrete (**a**) compressive strength and (**b**) peak strains.

Square and rectangular sections were used in this study to evaluate the efficacy of hemp and cotton strengthening. In experimental results, it was found that for the same number of layers, the enhancement in the compressive strength and the corresponding strain was predominant in square sections as compared to rectangular sections. To consider the shape of the rectilinear section in analytical models, the aspect ratio of rectilinear sections was included in the equations of ${k}_{A}$ and ${k}_{B}$ as given in Eqs. (12) and (13). The aspect ratio of a section is defined as the ratio of its longer side to the shorter side.

12 $${k}_{A}=A{\left(\frac{d}{b}\right)}^{a}{\left(\frac{{f}_{u}}{{f}_{co}}\right)}^{\alpha }$$

13 $${k}_{B}=B{\left(\frac{d}{b}\right)}^{b}{\left(\frac{{f}_{u}}{{f}_{co}}\right)}^{\beta }$$

where $d$ is the longer side and $b$ is the shorter side of the section. Nonlinear regression was carried out to estimate the constants $A, a, \alpha , B, b,$ and $\beta$. These constants are summarized in Table 9. Figure 18 presents the comparison between the analytical and experimental compressive strength and peak strain for rectangular sections.

**Table 9:** Regression constants for rectangular sections.

Constant	Hemp confinement	Cotton confinement
$A$	1.86	1.72
$a$	− 1.94	− 1.86
$\alpha$	− 0.54	− 0.52
$B$	25.38	19.41
$b$	− 0.41	− 1.06
$\beta$	− 0.32	− 0.42

##### Post-peak modulus for cotton confinement

As stated earlier, the current study limited the prediction of stress–strain curves of rectilinear sections to cotton confinement only. It is interesting to note that the rate of strength degradation increased in rectangular sections, as compared to square sections, where the corresponding values are presented in Table 10. Therefore, different regression coefficients were proposed. In addition, the rate of strength decay decreased as the number of cotton layers increased. As a result, the confinement pressure was included in regression analysis as one independent variable, whereas the aspect ratio was included to account for different strength decay rate in square and rectangular sections. The following Eq. (14) was proposed from regression analysis with a coefficient of determination of 0.98.

**Table 10:** Comparison of post-peak modulus for cotton confinement on square and rectangular sections.

Number of layers	Post-peak modulus (MPa)	
	Square	Cotton
1	− 271.19	− 699.60
2	− 180.25	− 316.09
3	− 53.48	− 160.84

14 $$Z=-1795.24{\left(\frac{d}{b}\right)}^{1.27}{\left({f}_{u}\right)}^{-1.24}$$

##### Experimental and predicted stress–strain curves: comparison

**Figure 19:**
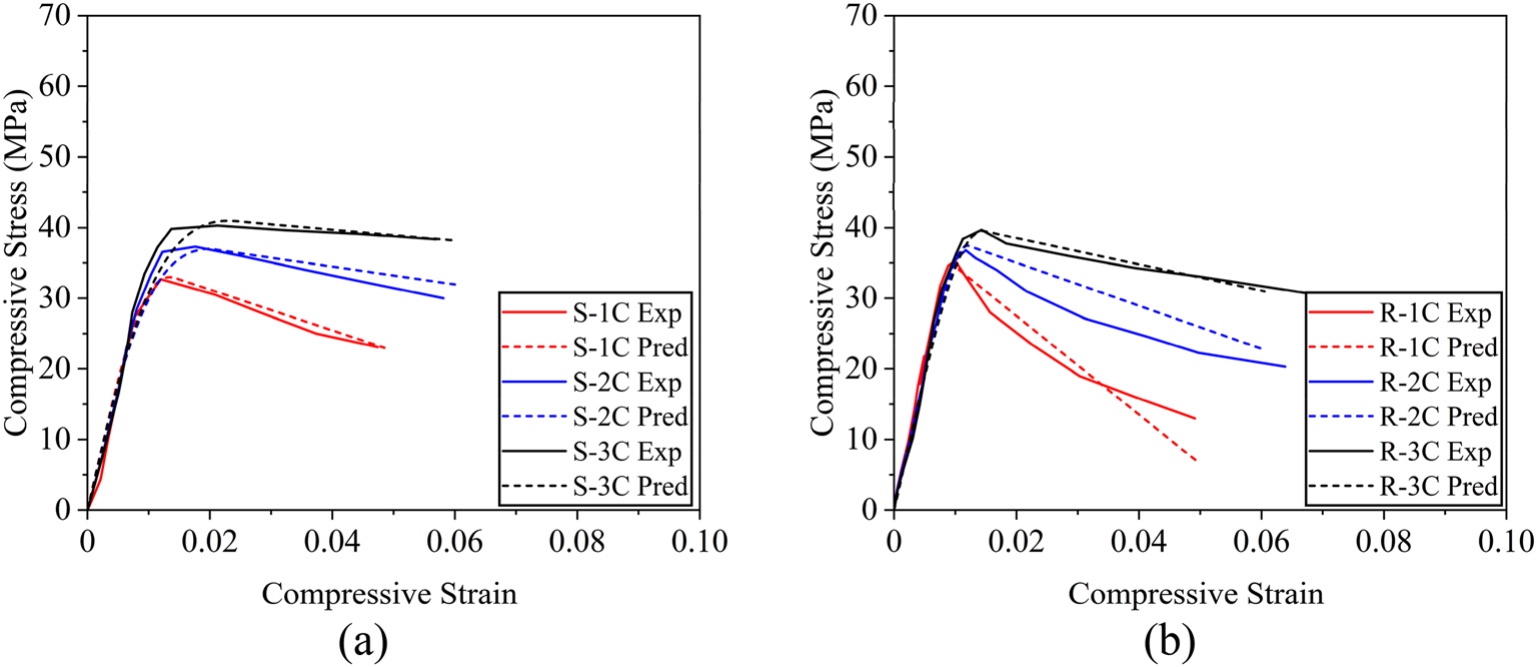
Comparison of stress strain curves for cotton confined concrete (**a**) square section and (**b**) rectangular section.

Figure 19 demonstrates the comparison between the experimental (Exp.) and predicted (Pred.) stress–strain curves. The stress–strain curves predicted in the analysis are found to correlate well with the experimental results. An exclusion was noticed in the case of Specimen R-2C, where the post-peak strength degradation was underestimated.

## Conclusions

This study involved both experimental and analytical investigations into the effectiveness of natural fiber rope reinforcement polymers (FRRP) in improving the compressive strength and ductility of reinforced concrete. To this end, a total of 36 specimens were tested, divided into three groups of twelve specimens each. The groups consisted of circular, square, and rectangular specimens. Natural FRRP made from hemp and cotton fiber ropes were applied in 1, 2, and 3 layers for each group. Depending on experimental findings, several significant inferences can be made. It is important to note that the following conclusions were drawn from axial compression tests performed on small-scale cylinders and rectilinear specimens. The findings of this study need proper attention while extrapolating to specimens of different sizes, especially when the aspect ratio of rectangular specimens changes. Moreover, this study utilized a constant corner radius of 13 mm. A larger corner radius would have improved the efficiency of the proposed FRRP further. Therefore, further studies are needed to explore the relationship between the size of the corner radius and the efficiency of FRRP confinement.

1. Both the cotton and hemp FRRP were able to improve the compressive behavior of the reinforced concrete. For circular specimens, a three-layer hemp FRRP confinement improved the compressive strength and corresponding strain by 200% and 270%, respectively, whereas the corresponding improvement imparted by the cotton FRRP was 117% and 233%, respectively. A three-layer hemp FRRP on square specimens improved the compressive strength and the corresponding strain by 110% and 186%, respectively, whereas similar cotton confinement brought these improvements by 95% and 144%, respectively.
2. The unconfined specimens showed a sudden drop in the compressive stress–strain relation after reaching the compressive strength, while the strengthened specimens exhibited a ductile behavior in their post-peak response. The degrading modulus of the post-peak branch improved as the number of FRRP layers increased. Furthermore, a bilinear compressive stress versus strain behavior was observed for the specimens confined with hemp FRRP.
3. Maximum enhancement in the compressive response of the reinforced concrete was observed for the case circular sections followed by square and rectangular sections, respectively. Further, for the same number of FRRP layers, hemp confinement induced a greater increase in the compressive strength and the corresponding strain than the cotton confinement.
4. The existing models could not estimate the mechanical properties of natural FRRP confined reinforced concrete with reasonable accuracy. Hence, nonlinear regression was performed to propose analytical models for hemp and cotton confined circular, square, and rectangular section concrete to predict the compressive strength and the corresponding strain. A close agreement between experimental and analytically predicted mechanical properties was observed.
5. The study modified an existing modeling scheme to capture the stress–strain responses of RC specimens externally wrapped with hemp and cotton FRRP. The results obtained through the modified model were found to be in excellent agreement with the experimental results, demonstrating its efficacy in predicting the stress–strain curves of RC specimens externally wrapped natural FRRP.

### Supplementary Information

Supplementary Information.

## Data Availability

The datasets used and/or analysed during the current study are available from the corresponding author on reasonable request.

## Abbreviations

RC: Reinforced concrete
FRP: Fiber-reinforced polymer
FRRP: Fiber rope reinforced polymer
L: Length of bar between central stirrups
D: Dimeter of longitudinal reinforcement
AVG: The average value of the ratios of experimental to predicted values of compressive strength or ultimate strains
SD: Standard deviation
${E}_{c}$: Elastic modulus of concrete
${\epsilon }_{cc}$: Compressive strain at the end of initial parabolic branch of stress versus strain curve
${\epsilon }_{co}$: Ultimate strain of unconfined concrete
${\epsilon }_{cu}$: Ultimate strain of confined concrete
${f}_{cc}$: Compressive stress at the end of initial parabolic branch of stress versus strain curve
${f}_{co}$: Compressive strength of unconfined concrete in MPa
${f}_{cu}$: Compressive strength of confined concrete
$Z$: Post-peak modulus
