# Supplementary material for: Stress and strain relations of RC circular, square and rectangular columns externally wrapped with fiber ropes
Source: Sci Rep. 2024 Feb 20;14:4181. doi: 10.1038/s41598-024-54586-9 (PMC10879512; doi:10.1038/s41598-024-54586-9)
Supplement: Supplementary file 1 — Supplementary Information. [file 41598_2024_54586_MOESM1_ESM.docx]

**Appendix**

Richart et al. ^66^ proposed the following form to relate the compressive strength of confined concrete $f_{cc}$ and the peak strain $\epsilon_{cc}$ to the lateral confining pressure $f_{l}$.

| $f_{cc}=f_{co}\left( 1+k_{1}\frac{f_{l}}{f_{co}} \right)$ | (A1) |
| --- | --- |
| $\epsilon_{cc}=\epsilon_{co}\left( 1+k_{2}\frac{f_{l}}{f_{co}} \right)$ | (A2) |

where $k_{1}$ and $k_{2}$ are coefficients estimated by the regression on experimental data, $f_{co}$ is the compressive strength in an unstrengthened condition, and $\epsilon_{co}$ is the compressive strain corresponding to $f_{co}$. According to Pellegrino and Modena ^50^, the total confining pressure $f_{u}$ is computed as the sum of $f_{lf}$ and $f_{ls}$ reduced by the ratio $A_{cc}/A_{g}$, where $A_{cc}$ is the area of concrete core measured from outside the transverse reinforcement and $A_{g}$ is the gross area of the section.

| $f_{u}=f_{lf}+fl_{s}\frac{A_{cc}}{A_{g}}$ | (A3) |
| --- | --- |

It is established that the efficiency of exterior wraps is better on circular sections as compared to rectilinear sections ^48,50,60,67^. This is because the cross-section of a circular section is fully confined. On the contrary, the presence of sharp corners induces stress concentrations that result in the reduced efficiency of external wraps ^35^. The lateral confining pressure by the FRRP sheet is related to the external confinement by Eq. (A4).

| $f_{lf}=\frac{2f_{t}t_{f}n_{f}}{D}\times k_{ef}$ | (A4) |
| --- | --- |

where $f_{t}$ is the rupture capacity of FRRP, $t_{f}$ is the thickness of FRRP, $n_{f}$ is the number of FRRP layers applied, $D$ is the size of the section, and $k_{ef}$ is a factor that accounts for the reduced efficiency of FRRP wraps due to corners. For a circular section, $D$ is its diameter, whereas the $D$ is replaced by an equivalent value defined by Eq. (A5) and shown in Figure A1 ^35^.

| $D=\frac{2bd}{b+d}$ | (A5) |
| --- | --- |

where $b$ is the width and $d$ is the depth of the section.





**Figure A1**. Description of bursting pressure and forces in FRRP sheet

The coefficient $k_{ef}$ is defined as the product of $k_{vf}$ and $k_{hf}$, where $k_{vf}$ is a factor that considers the reduced efficiency of FRRP wraps along the height of the specimen. For continuous FRRP wraps as used in this study, the term $k_{vf}$ is taken as 1.0. The factor $k_{hf}$ considers the reduced efficiency of FRRP wraps within a section. For a circular section, the factor $k_{hf}$ is taken as 1.0, whereas $k_{hf}$ is calculated using Eq. (A6) ^60,65^.

| $k_{hf}=\frac{1-\frac{w_{x}^{2}+w_{y}^{2}}{3bd}}{1-\rho_{cc}}$ | (A6) |
| --- | --- |

where $w_{x}$ and $w_{y}$ are defined in Figure 16a and $\rho_{cc}$ is the volumetric ratio of the longitudinal reinforcement. The definition of the confinement pressure due to internal transverse reinforcement is adopted from Mander et al. ^65^, defined in Eq. (A7) and illustrated in Figure A2.

| $f_{ls}=\frac{1}{2}\left( k_{es}\rho_{st}f_{yt} \right)$ | (A7) |
| --- | --- |

where $\rho_{st}$ and $f_{yt}$ are the volumetric ratio and yield strength of transverse reinforcement, respectively. The coefficient $k_{es}$ defines the reduction in the efficiency of transverse reinforcement due to sharp corners in rectilinear sections and the discontinuity of transverse reinforcement along the height of the specimen and defined as:

| $k_{es}=k_{vs}\times k_{hs}$ | (A8) |
| --- | --- |

where $k_{hs}$ is a factor that considers the ineffectively confined areas within a section. For circular sections, $k_{hs}$ is taken as 1.0, whereas $k_{hs}$ is defined in Eq. (A9) and shown in Figure 16b for rectilinear sections ^50,60,65^. Similarly, the parameter $k_{vs}$ incorporates the ineffectively confined areas along the height of the specimen, as defined in Eq. (A10).

| $k_{hs}=\frac{1-\sum\frac{w_{xi}^{2}+w_{yi}^{2}}{6xy}}{1-\rho_{cc}}$ | (A9) |
| --- | --- |
| $k_{vs}=\frac{\left( 1-\frac{s^{'}}{2x} \right)\left( 1-\frac{s^{'}}{2y} \right)}{1-\rho_{cc}}$ | (A10) |

where $\rho_{cc}$ is the longitudinal reinforcement volumetric ratio, $s'$ is the clear distance between consecutive hoops, and $x$ and $y$ are defined in Figure A2b.


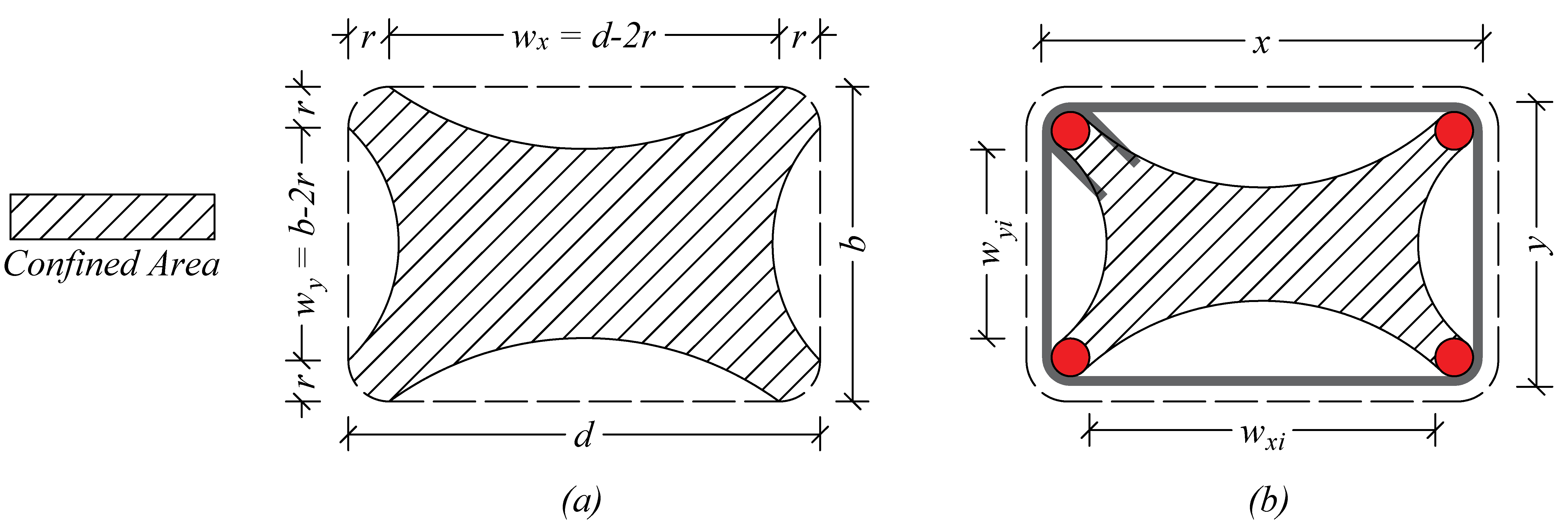


**Figure A2**. Definition of confined areas along a rectangular section (a) by FRP and (b) by internal steel reinforcement.
